# Determinants of corrosion resistance of Ti-6Al-4V alloy dental implants in an *In Vitro* model of peri-implant inflammation

**DOI:** 10.1371/journal.pone.0210530

**Published:** 2019-01-31

**Authors:** Larissa O. Berbel, Everson do P. Banczek, Ioannis K. Karousis, Georgios A. Kotsakis, Isolda Costa

**Affiliations:** 1 Instituto de Pesquisas Energéticas e Nucleares, Centro de Ciência e Tecnologia de Materiais, São Paulo-SP, Brazil; 2 Universidade Estadual do Centro-Oeste, Unicentro, Department of Chemistry, Guarapuava-PR, Brazil; 3 National and Kapodistrian University of Athens, Department of Periodontics, Athens, Greece; 4 Department of Periodontics, University of Texas Health Science Center at San Antonio, San Antonio, Texas; University of Vigo, SPAIN

## Abstract

**Background:**

Titanium (Ti) and its alloys possess high biocompatibility and corrosion resistance due to Ti ability to form a passive oxide film, i.e. TiO_2_, immediately after contact with oxygen. This passive layer is considered stable during function in the oral cavity, however, emerging information associate inflammatory peri-implantitis to vast increases in Ti corrosion products around diseased implants as compared to healthy ones. Thus, it is imperative to identify which factors in the peri-implant micro-environment may reduce Ti corrosion resistance.

**Methods:**

The aim of this work is to simulate peri-implant inflammatory conditions *in vitro* to determine which factors affect corrosion susceptibility of Ti-6Al-4V dental implants. The effects of hydrogen peroxide (surrogate for reactive oxygen species, ROS, found during inflammation), albumin (a protein typical of physiological fluids), deaeration (to simulate reduced pO_2_ conditions during inflammation), in an acidic environment (pH 3), which is typical of inflammation condition, were investigated. Corrosion resistance of Ti-6Al-4V clinically-relevant acid etched surfaces was investigated by electrochemical techniques: Open Circuit Potential; Electrochemical Impedance Spectroscopy; and Anodic Polarization.

**Results:**

Electrochemical tests confirmed that most aggressive conditions to the Ti-6Al-4V alloy were those typical of occluded cells, i.e. oxidizing conditions (H_2_O_2_), in the presence of protein and deaeration of the physiological medium.

**Conclusions:**

Our results provide evidence that titanium’s corrosion resistance can be reduced by intense inflammatory conditions. This observation indicates that the micro-environment to which the implant is exposed during peri-implant inflammation is highly aggressive and may lead to TiO_2_ passive layer attack. Further investigation of the effect of these aggressive conditions on titanium dissolution is warranted.

## Introduction

Titanium (Ti) and its alloys is commonly used for manufacturing dental implants owing to their high biocompatibility and corrosion resistance. These favorable properties for *in vivo* implantation are due to Ti’s ability to form a passive oxide film, i.e. TiO_2_, within seconds after exposure to oxygen that makes it bioinert [1–2]. Even if the passive oxide is broken, it can be rapidly regenerated in the presence of oxygen, leading to protection of the metal surface [3–4]. This passive layer is very stable during function even under demanding mechanical and chemical conditions, such as during mastication and exposure to fluids in the oral cavity, thus protecting the titanium surface from corrosion [5]. However, up to 20% of individuals with dental implants develop peri-implantitis; a chronic inflammatory disease that causes loss of jawbone around dental implants and eventually implant failure and removal [6].

The etiology of peri-implantitis has not been fully unraveled but has been traditionally considered to be associated to many causes, such as bacterial biofilm [7], corrosion [2,7], excessive mechanical stress [2], previous periodontal disease [7] and lesions of peri-implant attachment [2], besides excess cement [8]. Notably, recent results have demonstrated that Ti corrosion products are vastly increased around diseased implants as compared to healthy ones [9]. This observation may indicate that the micro-environment to which the implant is exposed during peri-implant inflammation is highly aggressive leading to TiO_2_ passive layer attack. In fact, a 2015 *ex vivo* microscopic investigation of peri-implant tissues revealed that peri-implant inflammation was characterized by the presence of foreign bodies, such as Ti particles [8]. Importantly, the authors stated that it is crucial to determine mechanism of introduction of these foreign bodies in peri-implant tissues, which is currently unknown [8].

Implant failure and removal have been associated with many factors, such as, corrosion of materials, presence of microorganisms, periodontal infections, mechanical stress and design of implant-abutment [10–12]. Various studies called attention to the effects of some properties of the environment in which the metal is inserted on the corrosion resistance of dental implants. Examples of the medium factors that affect the corrosion behavior of dental materials are microbiological and enzymatic characteristics [7,13], humidity [13], saliva composition [13], contact with blood and its constituents [14], biofilm formation [7,15], presence of fluorine and hydrogen peroxide [12,15], pH change in the buccal region [12, 15, 16], physical and chemical properties of foods [16], hypersensitivity reactions to titanium [15], presence or absence of oxygen [15], contact with different organic compounds and different concentrations of proteins [14,16].

Data exist to demonstrate that the Ti dissolution products that occur during Ti corrosion in biologic environments may affect the structure and composition of the submucosal microbiome towards a less diverse, more anaerobic community as well as contribute to inflammation via TLR-mediated recognition [17–18]. Therefore, there is a heightened interest in identifying factors triggering Ti corrosion during peri-implant inflammation. It is known that following implantation, titanium surfaces tends to adsorb proteins present in the environment [14]. In standard neutral pH conditions, proteins are generally negatively charged, while titanium is positively charged, leading to formation of colloidal organometallic complexes. Products generated in this interaction increase the metal dissolution, that is, its corrosion rate [14]. In fact, the corrosion behavior of Ti and its alloys in the presence of albumin [14, 19–20] is altered, usually leading to accelerated oxidation. Further, Taman and Turlyilmaz [21] studied the electrochemical behavior of Ti implants in Ringer's physiological solution with pH 5.5 and 7.0, either without or with dextrose simulate diabetic condition and higher susceptibility to corrosion was found in solution of pH 5.5 with dextrose. Ti samples tested in Ringer’s physiological solutions of pH 7.0 without dextrose showed very low passive densities (approximately 0.24 μA/cm^-2^). EIS measurements showed that acidic environments resulted in decreased impedances and SEM observation indicated that environments of low pH and high glucose concentration are associated to corrosion.

To investigate the determinants of Ti corrosion resistance, an *in vitro* model of peri-implant inflammation utilizing clinically-relevant acid-etched Ti-6Al-4V (grade V) alloy discs was developed. The core of the experimental model included a three-electrode cell with an isotonic fluid as the corrosive medium. Additionally, we employed the use of hydrogen peroxide as a surrogate for reactive oxygen species (ROS) found during inflammation, albumin (a protein typical of physiological fluids), deaeration to simulate reduced pO_2_ conditions during inflammation, and lowering of pH to resemble acidic environmental conditions during inflammation. The hypothesis for this study was that in low pO_2_ environments re-passivation of titanium is hindered, thus reducing its corrosion resistance. In order to characterize the electrochemical properties of titanium under peri-implant-inflammatory conditions for the first time, interactions with bacterial biofilms were not included in this study. The present study focused on conditions typical of crevice environments and inflammation on the protective properties of the titanium oxide “passivation” film responsible for the corrosion resistance of Ti alloys used in dental implants. The sequential investigation of the key components that determine electrochemical behavior of titanium in inflammation, i.e. pH, protein concentration, oxygen radicals and aeration conditions, is novel.

## Materials and methods

Specimens for this study were fabricated with Ti-6Al-4V alloy (Titanium grade V) in 10 mm diameter disks. The discs were acid-etched to simulate clinically relevant procedures by a proprietary acid-etching method following the same protocol for commercial dental implant surface preparation (Zuga medical, OH, US). The chemical composition of the alloy obtained by X-ray fluorescence analysis (XRF) is presented in Table 1.

**Table 1:** Chemical composition (wt.%) of the Ti-6Al-4V alloy used in this study.

Element	Ti	Al	V	Cr	Mn	Fe	Cu	Nb	Sn
**wt. %**	Bal.	7.31	4.14	-	0.01	0.18	0.01	0.02	0.01

Prior to all experimentations the surfaces of the discs were sonicated in ethanol for 10 minutes, for removal of contaminants, rinsed with deionized water and dried in hot air stream and finally immersed in phosphate buffered solution (PBS) with pH adjusted to 3 with phosphoric acid.

### *In vitro* model of peri-implant inflammation

**Fig 1:**
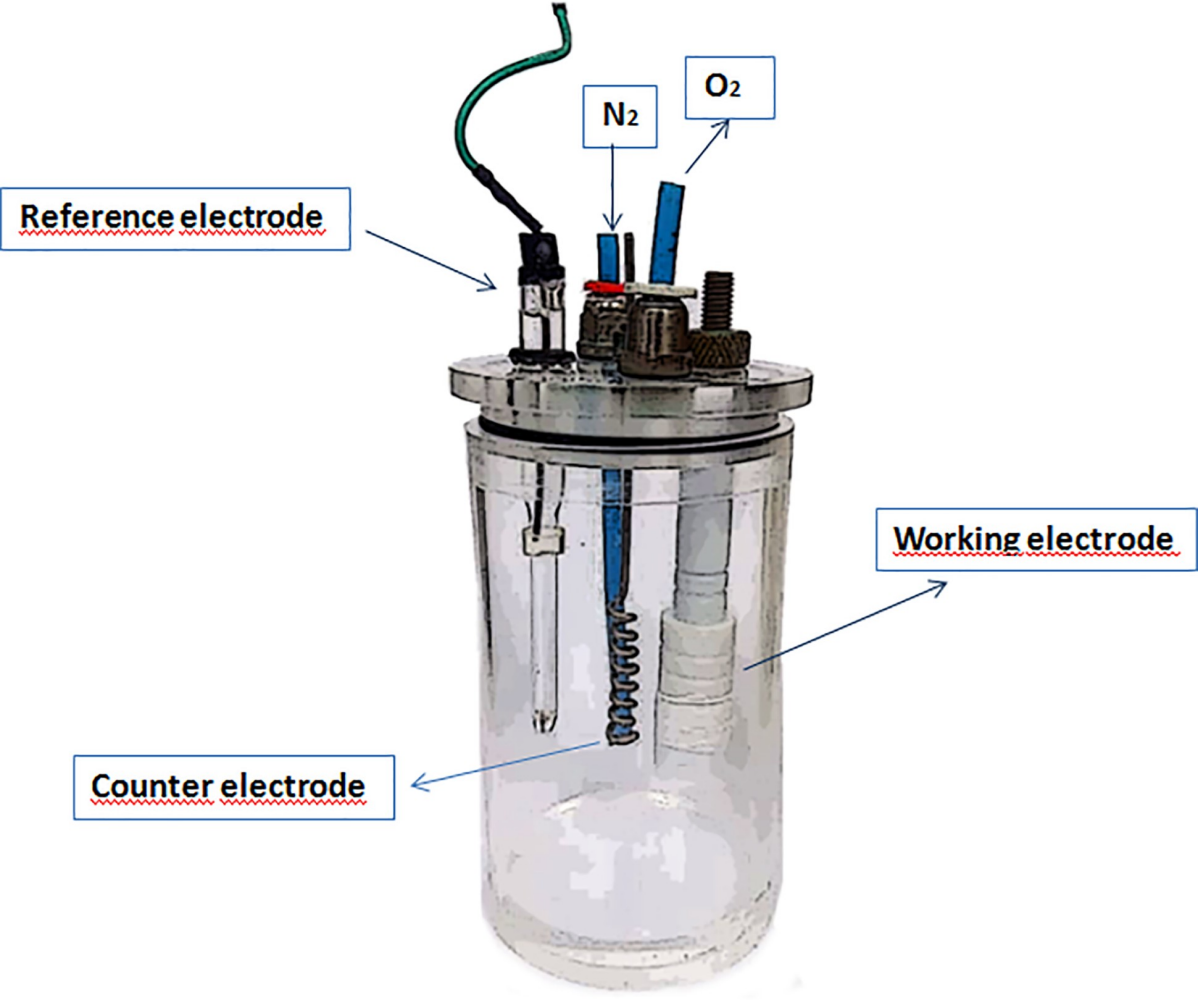
Schematic illustration of the three-electrode electrochemical cell used in this study.

Simulation of peri-implantitis *in vitro* requires rendering of environmental conditions that occur in the peri-implantitis tissues during inflammation or peri-implantitis treatment procedures. These conditions may affect titanium electrochemistry. Peri-implant inflammation is characterized by a vast increase in reactive oxygen species, a.k.a ROS due to the ongoing inflammatory process (e.g. influx of polymorphonuclear granulocytes) as well as oxidative stress [22]. The model utilized in the present study was composed of a three-electrode cell with an isotonic fluid (electrolyte) as the corrosive medium. The three-electrode cell was used for electrochemical measurements with an Ag/AgCl (3M KCl) reference electrode, a platinum wire as counter electrode and the Ti-6Al-4V discs as working electrodes (Fig 1). Following placement of the titanium specimens in the cell, stepwise exposure to the following environmental conditions was performed to better simulate relevant clinical scenarios:

- Deaeration was employed to simulate conditions inside occluded areas where oxygen depletion occurs, which are conditions typical of crevice corrosion [23]. Further, deepening of the sulcus as part of the disease process is associated to reduction in oxygen tension [24]. Deaeration simulates reduced pO_2_ conditions during inflammation and in the cell it was achieved by purging of the isotonic solution with nitrogen for 20 minutes prior to electrochemical tests. Acidic conditions were induced to local acidification inside occluded areas as well as acidic pH environment relating to food challenges and inflammation. Lowering of pH to resemble acidic environmental conditions during inflammation was carried out with addition of phosphoric acid. The PBS solution used in this study was acidified to pH 3 by adding phosphoric acid with the aim of simulating the acidic conditions typical of crevices and inflammation. Phosphoric acid was chosen for pH change to maintain the same type of anions already present in the PBS solution, i.e., phosphate ions.
- Hydrogen peroxide (H_2_O_2_) 1% (wt.) was employed as a surrogate for ROS during peri-implant inflammation [25]. ROS are key molecules that are abundant in inflammation and characterize the aggressive environment that surrounds titanium during inflammation in peri-implantitis [22]. Previous work [26] investigated the effect of peroxide concentration in the range from 0–10% on the corrosion resistance of a Ti-alloy. The authors [26] found that the addition of 10% of H_2_O_2_ was too aggressive towards the Ti surface resulting in the formation of complexes Ti- H_2_O_2_ that modified the amount of Ti dissolved in the solution. On the other hand, 1% hydrogen peroxide provided the oxidizing effects to the environment without leading to overly aggressive conditions to the solution. Based on the literature [24], in this work a 1% concentration of H_2_O_2_ was adopted.
- Albumin 1% (wt.) [26, 27], a protein typical of physiological fluids and one of the most abundant proteins in plasma, was also utilized as it modifies colloidal osmotic pressure locally [28]. The concentration was selected because according to existing literature [27] albumin composes approximately 1% of salivary proteins. The aforementioned factors were assessed sequentially to resemble local environmental conditions during peri-implant inflammation.

### Electrochemical tests

Electrochemical tests used in this study were open circuit potential (OCP) measurements as a function of time of exposure to test solution, electrochemical impedance spectroscopy (EIS) and anodic polarization tests. Fig 1 shows a schematic diagram of the electrochemical cell used in the electrochemical tests used. After polarization the surface of Ti-6Al-4V was observed by scanning electron microscopy (SEM) with 1 wt. % BSA and hydrogen peroxide in aerated and deaerated conditions.

EIS measurements were carried out using a frequency response analyzer (Gamry Reference 600+), coupled to a potentiostat (Gamry PC600P) with an applied potential sine-wave perturbation of 10 mV_rms_ in the frequency range from 50 kHz to 10 mHz using an acquisition rate of 8 points per frequency decade. Anodic polarization curves were obtained after EIS tests with a scan rate of 1 mV s^-1^ starting from OCP up to 2.0 V vs Ag/AgCl (3M KCl). OCP measurements were also carried out for 10 minutes after EIS test termination. All the tests were carried out at 25 ^o^C after 3h of exposure to the various electrolytes tested. The electrochemical tests were performed in triplicate for evaluation of results reproducibility. Previous work carried out by Assis [29] with the Ti-13Nb-13Zr alloy in 0.9% NaCl solution showed no significant effect of temperature variation between 25 and 37 ^o^C in the alloy corrosion behavior.

## Results

### Effect of deaeration on electrochemical results

**Fig 2:**
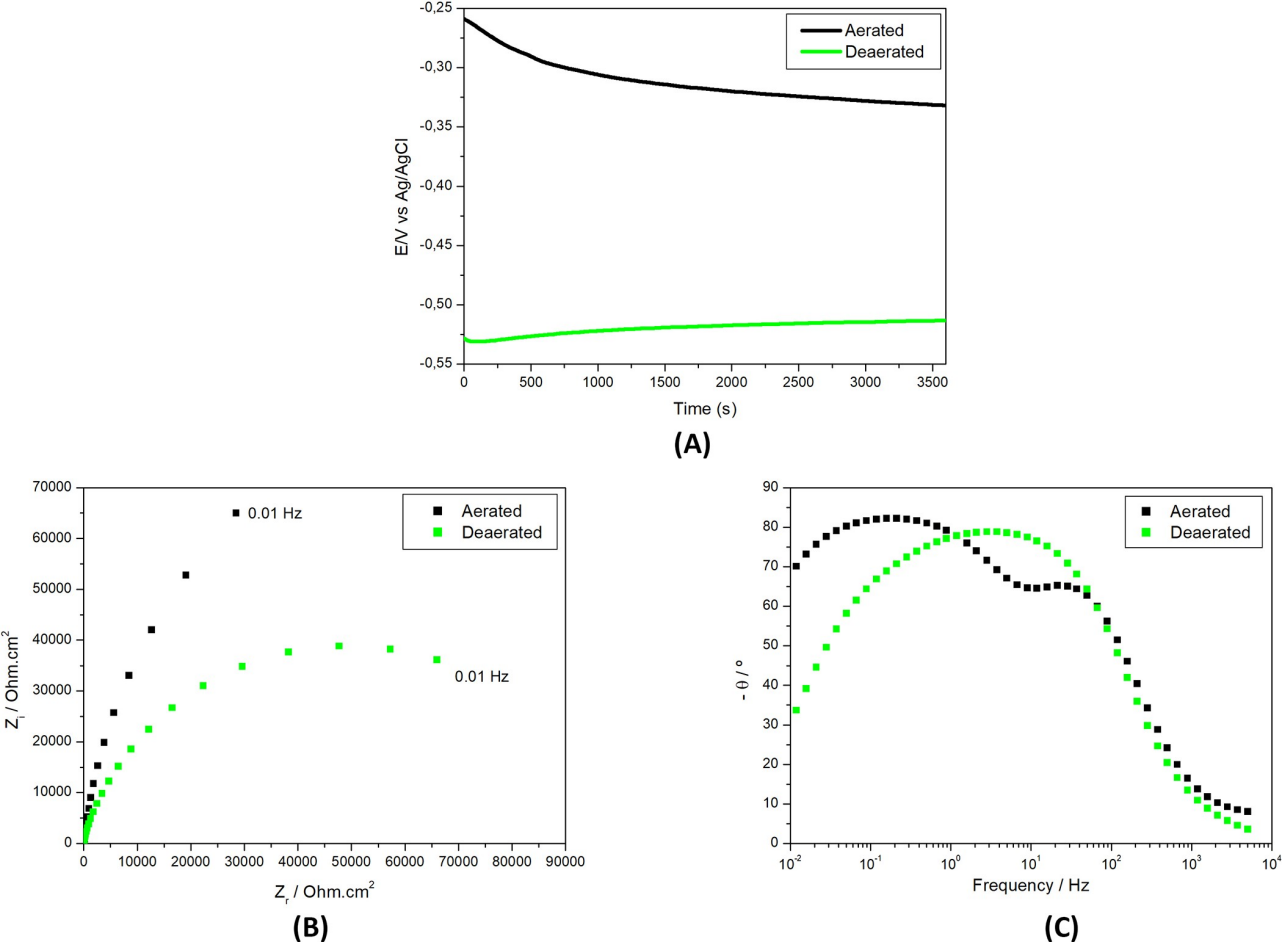
Electrochemical results of Ti-6Al-4V alloy in PBS solution (pH 3) (A) Open circuit potential (E) variation with time of immersion and electrochemical impedance spectroscopy (EIS) as (B) Nyquist and (C) Bode phase angle diagrams in deaerated or naturally aerated solutions.

Fig 2 shows the effect of deaeration on the electrochemical behavior of Ti alloy in the PBS with pH 3 by showing OCP and EIS results in aerated and deaerated solutions.

The effect of deaeration was clearly seen by the differences in OCP in the two solutions, aerated and deaerated. Nobler potentials were measured in the aerated during all the test duration. Initially the differences in potential were in the range of 0.27 V, that is, a significant difference in potential between the two conditions, aerated and deaerated. After 1 h of exposure to the two types of solutions, the differences in OCP decreased to around 0.18 V, still high enough to promote galvanic coupling effects if the same material is exposed to differential aeration. This shows that the availability of oxygen as oxidizing agent has a significant effect on the corrosion resistance of the Ti-alloy by favoring the restoration of oxide and, consequently, maintaining the Ti-alloy surface protection. This observation is supported by EIS results (Fig 2B) that showed higher impedances in aerated environment compared to deaerated. In the aerated solution, two-time constants were clearly distinguished in the Bode phase angle diagrams. In the published literature a model of double layer structure for the passive film on Ti and Ti alloys has been proposed, composed of an inner and compact layer and an outer and porous layer [30–32]. A two-time constant process was only clearly observed for the EIS results of the present study obtained in aerated solution with a first time constant at approximately 100 Hz being related to the oxide film on the Ti alloy surface and the second-time one at frequencies from 1 to 0.1 Hz associated to charge transfer processes at the substrated exposed on the defective areas of the oxide coupled to charging of the double layer. There is also indication of interaction of time constants in the deaerated solution meaning they are close to each other and, consequently, are not clearly separated. The lower impedances obtained in deaerated condition show that oxygen availability is very important for corrosion protection of Ti surfaces. The large difference in OCP values between the two environments suggests that galvanic coupling might occur in situations where Ti alloy surfaces are exposed to both conditions, partially exposed to environment where oxygen finds easy access to the surface (aerated) and partially to milieu of hard oxygen admittance (deaerated). This results in effective galvanic cells being formed with potential differences between anodic (deaerated) and cathodic (aerated) areas. The results also show that attack of the oxide film is favored in the acid PBS solution under deaerated condition.

### Effect of peroxide on electrochemical results

**Fig 3:**
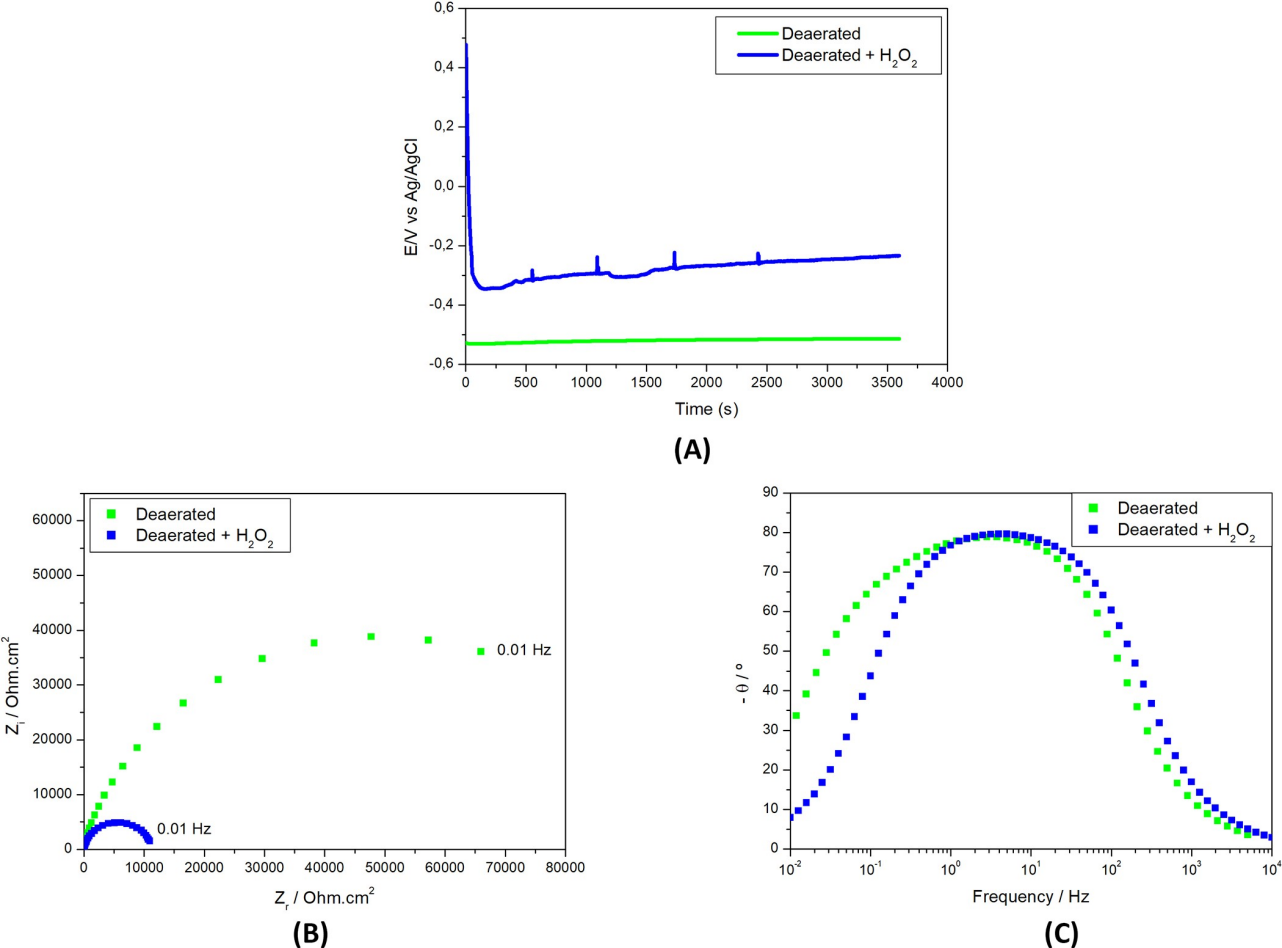
Electrochemical results of Ti-6Al-4V alloy in deaerated PBS solution (pH 3) (A) Open circuit potential (E) variation with time of immersion and electrochemical impedance spectroscopy (EIS) as (B) Nyquist and (C) Bode phase angle diagrams in solutions without 1 wt. % H_2_O_2_ or with 1 wt. % H_2_O_2_.

The effect of hydrogen peroxide (H_2_O_2_) addition of into the deaerated PBS solution is shown in Fig 3.

The oxidizing effect of this additive is shown by the higher OCP values measured compared to the solution without it since the first periods of test. Hydrogen peroxide is a strong oxidant and, as such, could promote oxide growth on titanium alloys surface but it could also result in the attack of the oxide formed by exposure to oxygen. The results suggested that H_2_O_2_ had a harmful effect in the oxide film at the surface. OCP decreased with time of test and presented spikes corresponding to sudden variations which are indicative of corrosive attack of the environment to the exposed surface. OCP variations were followed by potential stabilization resulting from balance between oxide film attack and its repair. The indication of the prejudicial effect of H_2_O_2_ is supported by EIS results that showed lower impedances for the surface in the solution with peroxide.

### Effect of protein (BSA) on electrochemical results

**Fig 4:**
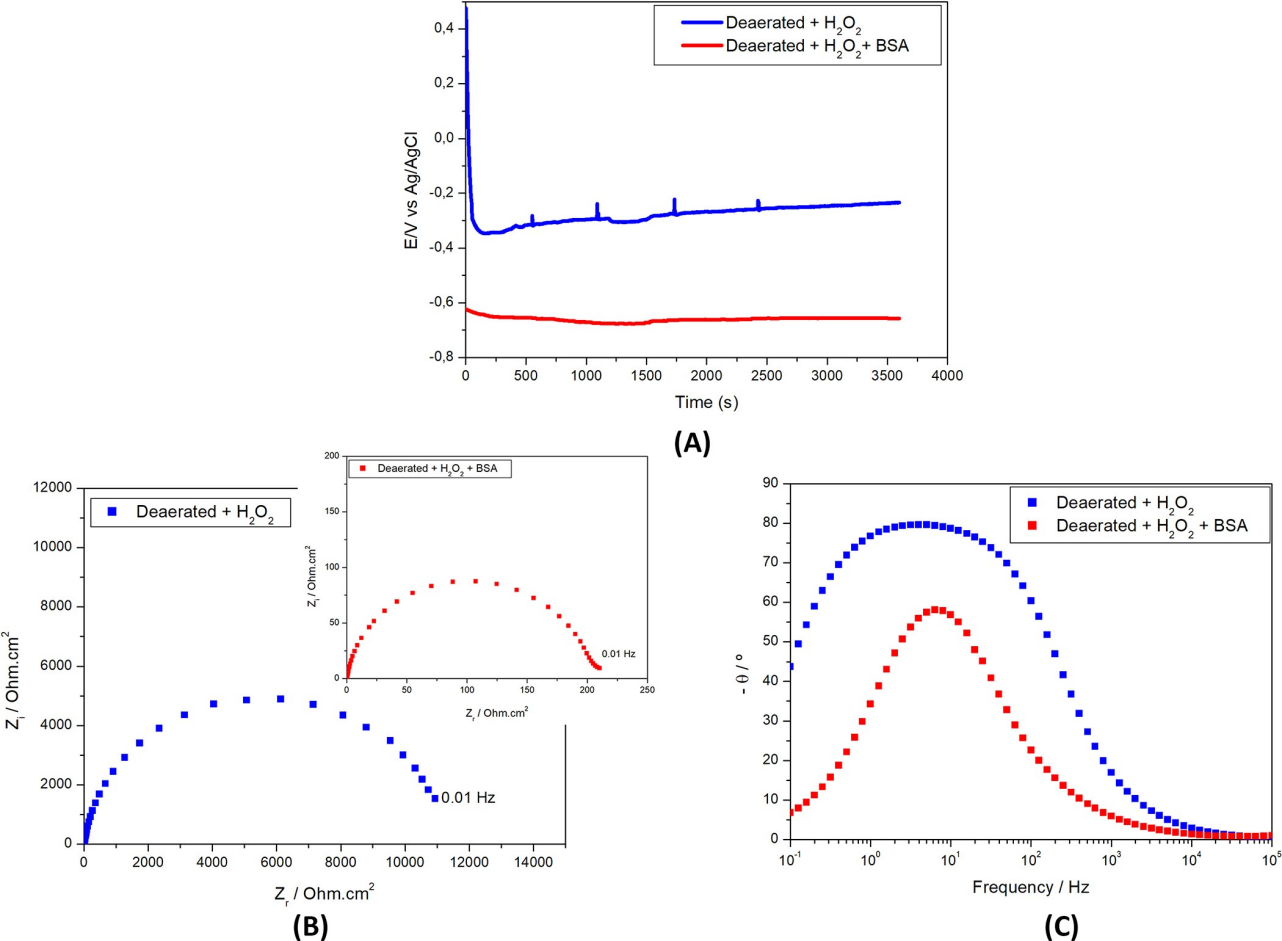
Electrochemical results of Ti-6Al-4V alloy in deaerated PBS solution (pH 3) (A) Open circuit potential (E) variation with time of immersion and electrochemical impedance spectroscopy (EIS) as (B) Nyquist and (C) Bode phase angle diagrams in solutions with 1wt. % H_2_O_2_ without BSA or with 1 wt. % H_2_O_2_ and 1 wt.% BSA.

Since proteins are normally found in the biological fluids its effect on corrosion resistance of the alloy used in dental implants was also investigated by adding 1 wt. % if BSA (fetal bovine serum) to the deaerated PBS solution (pH 3) with hydrogen peroxide addition and the results are presented in Fig 4.

OCP values in the solution with BSA rapidly decreased with time and were the most negative among the ones measured in the various solution tested. OCP stabilized after approximately 30 min of exposure indicating fast kinetics in this environment. This must be due to eased attack of oxide film in the solution with BSA. This result shows that the combination of low pH, deaeration, addition of H_2_O_2_ and BSA leads to a highly aggressive environment to the Ti alloy surface. This observation was supported by EIS results, (Fig 4(B) and (C)), that showed that the oxide film was no longer stable in this last solution. Addition of BSA to the test environment decreased impedance by approximately 10 times (Fig 4(B)). Besides, only one-time constant was seen in the Bode phase angle diagrams (Fig 4(C)) associated to charge transfer processes at the exposed metallic surface. However, for the solution without BSA, the Bode phase angle diagrams show a large and asymmetric peak indicative of interaction of two-time constants, associated to the oxide film and charge transfer processes at Ti alloy exposed to the test solution at the defective areas of the oxide film. These results show that under the conditions tested, BSA destabilizes the oxide film and promotes fast electrochemical activities of the Ti alloy by allowing its direct contact with the solution. These results contradict those of Karimi et al. [14] that found improved protective properties of the passive film on Ti in solutions with high concentrations of BSA.

### Effect of combination of conditions on polarization test results

**Fig 5:**
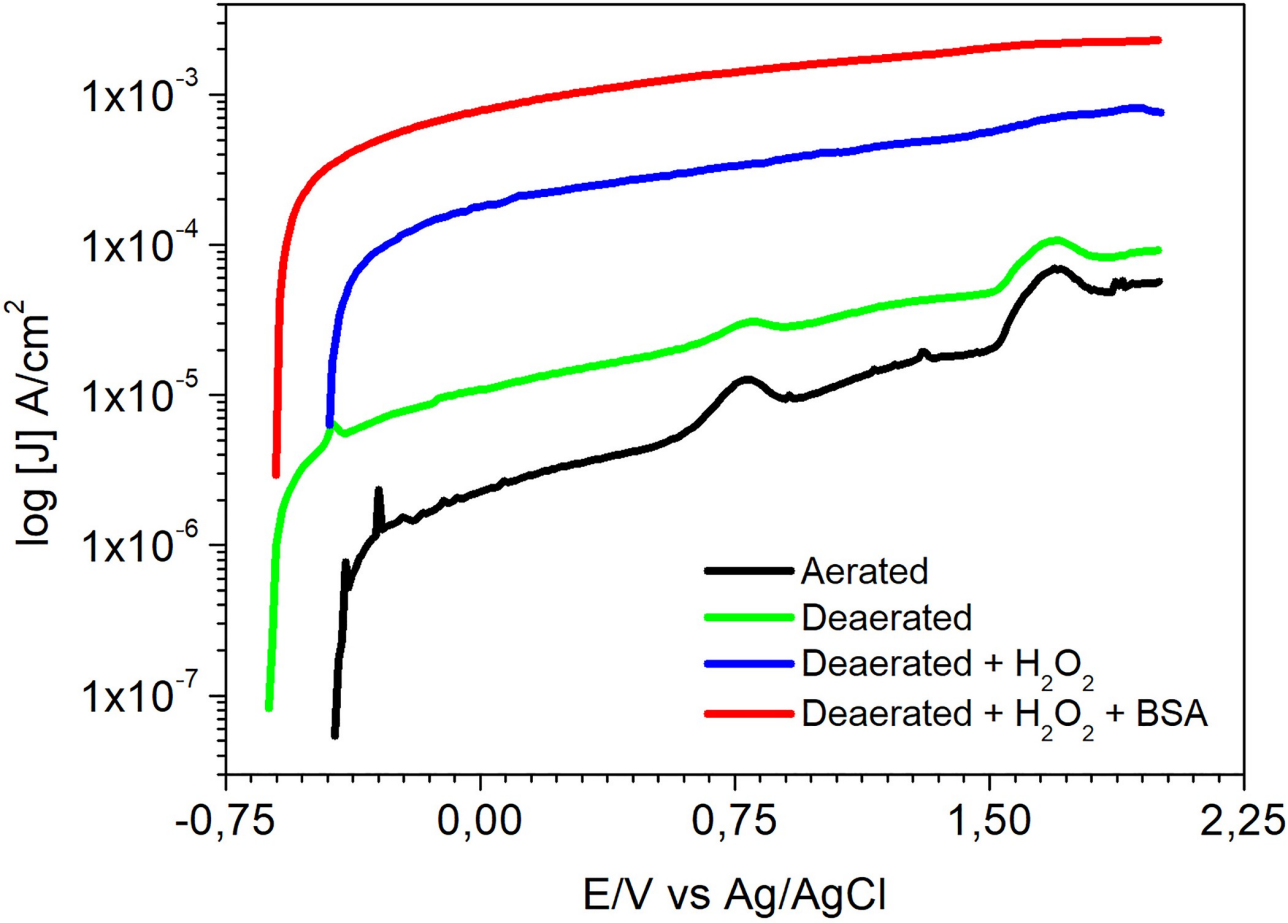
Anodic polarization curves of Ti-6Al-4V alloy in aerated PBS solution (pH 3), deaerated PBS solution (pH 3), deaerated PBS solution with 1 wt. % of hydrogen peroxide and deaerated PBS solution with 1 wt.% of hydrogen peroxide and 1 wt.% BSA.

Fig 5 shows the effect of various conditions tested in this study on the polarization results of the Ti alloy.

Current densities displayed in the polarization curves supported the results of OCP measurements and EIS. They largely increased with potential (*E*), in aerated or deaerated solutions, starting from 10^−6^ A/cm^2^. Current densities in the range from 10^−6^ A/cm^2^ to 10^−5^ A/cm^2^ are typical of passive films. As current densities increase above this range, there is indication of passive film attack by test solution. Values in the order of 10^−4^ A/cm^2^ or higher were reached in the both solutions, however higher current densities were related to deaerated condition. The defective oxide film resulting from its attack by the environment leads to higher current densities crossing the interface.

**Fig 6:**
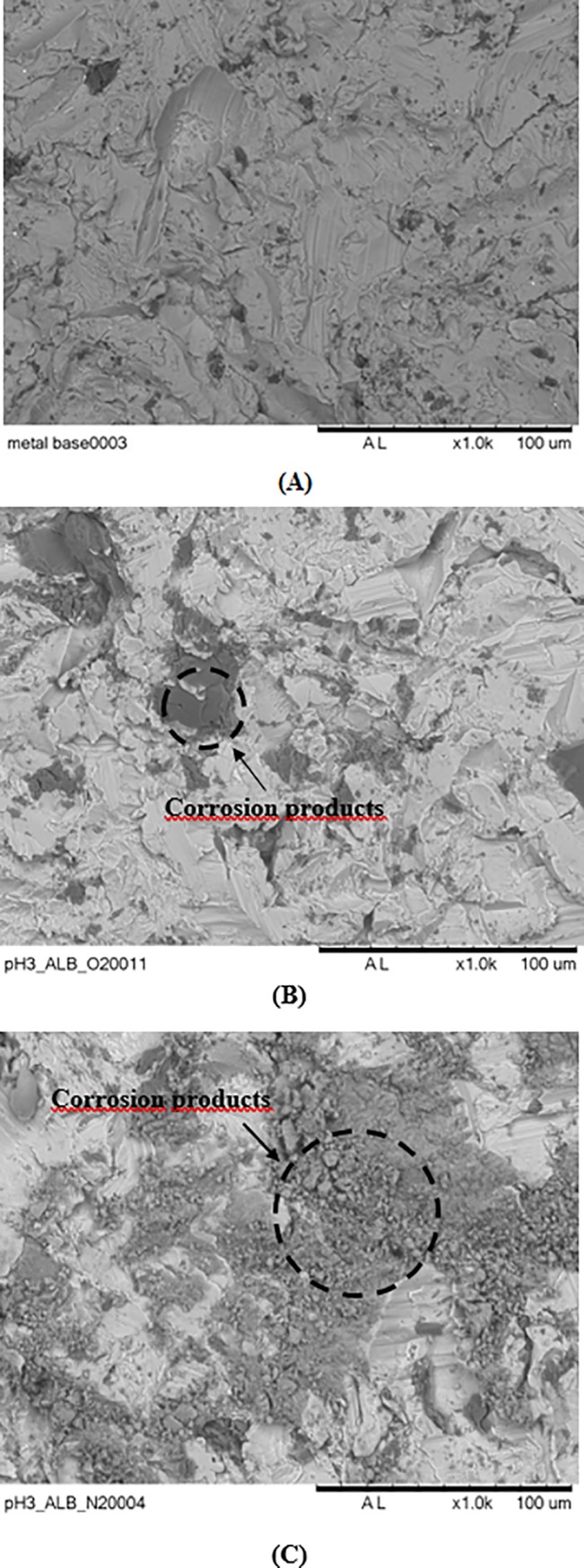
SEM images of Ti-6Al-4V sample as received and unexposed to the test solution (A), after polarization in PBS solution (pH 3) with 1 wt. % BSA and 1 wt. % of hydrogen peroxide, under naturally aerated condition (B) and same as (B) in deaerated condition (C).

Polarization curves in Fig 5 also show current density peaks occurring at similar potentials in both test solutions, aerated and deaerated. The first peak, at approximately -0.4V, is associated to the oxidation of Ti to Ti^3+^, according to Pourbaix diagram [33]. In solutions of pH 3 and at highly oxidizing potentials, Ti^3+^ is oxidized to Ti^4+^ leading to increase in current density and, subsequently, to TiO_2_ formation. The third current peak refers to the oxygen evolution reaction which is favored in aerated solutions. Despite the similarities seen in aerated and deaerated solution, higher current densities were obtained in deaerated environment showing lower resistance of the surface oxide film in this solution comparatively to that exposed to an environment with free oxygen access. Additions of 1 wt.% H_2_O_2_ to the solution and 1 wt.% of BSA promoted increase in current density, what is in agreement with the previous electrochemical results. Current density increased from values in the order of 10^−6^ A/cm^2^, that is, typical of passive films, and reached values in the order of 10^−4^ A/cm^2^, with H_2_O_2_ addition, and 10^−3^ A/cm^2^, when BSA was introduced in the test solution. These results show that the passive film is increasingly unstable in acidic and deaerated conditions when H_2_O_2_ and BSA are present in the environment. This is confirmed by SEM images in Fig 6 showing that the most aggressive environment to the Ti-6Al-4V was the deaerated acidic one (pH 3) in presence of albumin and hydrogen peroxide.

## Discussion

In the present study peri-implant inflammatory conditions were simulated *in vitro* and extensive electrochemical testing to determine factors affecting corrosion susceptibility of Ti-6Al-4V titanium surfaces were performed in phosphate solution. The major finding of the present investigation was the hazardous effects of reduced oxygen availability, hydrogen peroxide and protein (BSA) but mainly the combination of these factors on titanium’s passivity degradation. A distinct difference between the corrosion resistance of titanium in the presence versus absence of oxygen was noted across all experiments (results not presented here) irrespective of the additional experimental parameters. Assessment of various variables present in the implant microenvironment showed that the most aggressive conditions to the Al-6Al-4V alloy were these typical of occluded cells, *i*.*e*. oxidizing conditions (H_2_O_2_), high acidity (pH 3), presence of protein and deaeration of the physiological medium. Our results provide evidence that titanium corrosion can be triggered by intense inflammatory conditions.

The results showing harmful effects of BSA in the acid solution (pH 3) with H_2_O_2_ on the Ti-6Al-4V alloy corrosion resistance contradict some published literature. For instance, Khan et al. [34] analyzed the influence of proteins in different titanium alloys in phosphate buffered saline solutions with bovine albumin and 10% fetal calf serum with various pHs and protein concentrations. Their results showed that albumin addition to the electrolyte increased the corrosion resistance of Ti alloys, in most of the pH tested. However, the solutions used in the tests carried out by those authors were different from the ones used in the present study, once there was no addition of H_2_O_2_ and the amount of proteins used in their study was 10 wt.% whereas in this investigation 1 wt.% was adopted. It is important to emphasize that BSA effect is dependent on pH. Beneficial effects might occur by BSA interacting with Ti alloy surface by adsorption with consequent partial coverage of the surface, depending on environment pH.

Hydrogen peroxide is a powerful oxidant that can be found in the mouth region through its production by bacteria and in inflammatory processes. Mabilleau et al. [35] investigated the corrosion resistance of cp-Ti discs by atomic force microscopy (AFM) and scanning electron microscopy (SEM) after 9 days of immersion in different artificial saliva solutions containing fluoride, hydrogen peroxide and lactic acid. In the disks, bacteria were cultured in Dulbecco's modified Eagle's medium (DMEM) at 37° C in 5% CO_2_ humidified culture cell. Subsequently, the bacterium was isolated and identified as S. mitis. Surface analysis by AFM and SEM showed pitting signs in the disks exposed to solution containing 0.1 and 10% hydrogen peroxide. Although these authors did not find significant effects of increasing concentration of H_2_O_2_ on the corrosion resistance of Ti, in another work [26] the corrosion rates largely increased with H_2_O_2_ concentration. Additionally, in the combined presence of NaF, lactic acid (pH 4.5) and H_2_O_2_, the Ti corrosion was stronger than when each of the additives was tested separately. It must be taken into consideration that fluoride ions act as complexing agents for Ti. Thus, a synergism occurred by the combination of these three types of additives.

Al-Mobarak et al. [30] studied the electrochemical behavior of titanium and its alloys in artificial saliva containing hydrogen peroxide (H_2_O_2_) at pH 3 and pH 7.2. It was found that the presence of H_2_O_2_ led to decrease in corrosion resistance of Ti and its alloys. The OCP measurements showed increase in the potential with time to nobler values, what can be observed in this study for the samples immersed in aerated environment. This increase of potential suggests the growth of the oxide layer beneath the surface of the metal. The effect of peroxide of hydrogen is clearly shown by the electrochemical impedance (EIS), because in Nyquist plot of Ti at pH 7.2 and pH 3 relates the increase in the addition of hydrogen peroxide with the decrease of the diameter semicircle suggesting that the corrosion resistance decrease as increase the amount of peroxide hydrogen.

The effect of hydrogen peroxide in the oral environment on the corrosion resistance of Ti-cp was investigated by Fonseca García et al. [36] in solution of pH 5.2 to simulate inflammatory conditions. Their study was carried out by electrochemical methods, such as, open circuit potential, impedance spectroscopy and potentiodynamic polarization tests. Ti cp samples were immersed in a mixture of Hartman solution and 50 mM of hydrogen peroxide for 7 days. The electrochemical results indicated decreased corrosion resistance in solution with H_2_O_2._ Also, Ti surface analyses by atomic force microscopy (AFM), X-Ray photoelectron spectroscopy (XPS), wettability and surface energy showed that the samples immersed in simulated inflammatory conditions presented significant modifications in the Ti cp surface properties. PAN et al. [37] studied the enhanced oxide growth on titanium due to the presence of H_2_O_2_ by electrochemical impedance. They observed that in titanium immersed in PBS solution with H_2_O_2_ the passive film growth rate enhanced comparatively to the solution without peroxide, and the dissolution/oxidation resistance decreased due to two-layer structure oxide film.

When titanium contacts hydrogen peroxide, two corrosion phenomena occurs in the metallic surface, one is the decomposition of H_2_O_2_ catalyzed by the Ti surface and the other is the metal corrosion, that involves metal dissolution in electrolyte and the growth of titanium oxide [30,32].

The anodic half-cell for the metal dissolution is described as follow:

$$2Ti+O_{2}+H_{2}O\to Ti_{2}O_{3}+2H^{+}+2e^{‑}$$ (1)

and the cathodic half-cell in neutral and aerated conditions is mainly the oxygen reduction reaction, according to:

$$O_{2}+2H_{2}O+4e^{‑}\to 4OH^{‑}$$ (2)

In acidic and aerated environments, hydrogen ions reduction might also result in H_2_O (3) or H_2_O_2_ formation (4):

$$O_{2}+4H^{+}+4e^{‑}\to 2H_{2}O$$ (3)

$$O_{2}+2H^{+}+2e^{‑}\to H_{2}O_{2}$$ (4)

The peroxide promotes the formation of TiO_2_ by oxidation of a less stable oxide (Ti_2_O_3_) [38–40], leading to its decomposition as shown in (5):

$$Ti_{2}O_{3}+H_{2}O_{2}\to 2TiO_{2}+H_{2}O+2e^{‑}$$ (5)

However, in the presence of hydrogen peroxide, the oxygen and transition metal ions can form hydroxyl radicals increasing the electrolyte aggressiveness to the Ti alloys surface. The formation of hydroxyl radicals can be described by the reactions shown in Eqs (6) and (7). According to literature, Ti^3+^ acts as a Fenton reagent that interacts with H_2_O_2_ generating hydroxyl radicals and Ti^4+^ ions, according to (8), through the proposed mechanism and destabilizing the oxide film on the surface [38–40]:

$$Ti^{3+}+O_{2}⇆Ti^{4+}+O_{2}{}^{‑\cdot }$$ (6)

$$O_{2}{}^{‑\cdot }+2H^{+}\to O_{2}+H_{2}O_{2}$$ (7)

$$Ti^{3+}+H_{2}O_{2}\to Ti^{4+}+OH^{‑}+OH\cdot$$ (8)

In the presence of high oxygen contents, the equilibrium represented by (6) is displaced to the right, that is, Ti^3+^ oxidation and superoxide formation (O_2_^-^⋅), generating H_2_O_2_, that in turn, promotes more Ti^3+^ oxidation and hydroxyl radical formation. This favors TiO_2_ formation and growth increasing the oxide film protection. In the present study, the peaks of OCP variation corresponding to fast increase in potential followed by potential stabilization in this type of solution, suggests a competitive mechanism between the growth of the oxide and its attack resulting in an unstable condition.

Studies have evaluated the corrosion behavior of grade V titanium alloy in the presence of protein. Karimi et al. [14] studied the corrosion behavior of AISI 306L, Co-28Cr-6Mo and Ti-6Al-4V in solutions with different concentrations of bovine serum albumin (BSA) in phosphate buffered saline (PBS) solutions (pH 7.4). The lowest OCP was related to the highest concentration of BSA (4.0 g L^-1^). In solutions of lower concentrations (0.2–2.0 g L^-1^), however, OCP stabilized at higher potentials comparatively to solution without BSA. It is important to mention that protein concentrations up to 4.0 g L^-1^ are found in body fluids, and 4.0 g L^-1^ BSA concentration is typical of interstitial fluids [41]. The authors also found more stable potentials in solution with 4.0 g L^-1^ of BSA comparatively to solutions of lower concentrations and potentiodynamic polarization measurements showed that passive current densities decreased with BSA concentration from 0.2 to 4.0 g L^-1^ indicating a beneficial effect of BSA on the passivated surface of Ti alloy. These results suggest that BSA interaction with the metallic surface is dependent on its concentration in the environment. Albumin interacts with the metallic surfaces but the effect of the protein adsorption on the corrosion protection of the metallic substrate is dependent on its concentration in the environment [42–43]. In low concentrations, the adsorption can result in not effective protection of the substrate and the Ti oxidation reaction can be depolarized. Also, the inhibiting effect of this protein depends on the pH of the solution. In acidic media, the protein might be protonated and this alters its adsorption, even at high concentrations [43]. The total protein charge is related to the isoelectric point, that is, the total charge related to the protein is null. Below this point, the protein is positively charged, and above it, negatively charged. For albumin, the isoeletric point is in the pH range of 4.5–4.7 [44]. Consequently, in pH 3 the albumin is protonated and its adsorption is weak.

Polarization (Fig 5) results also showed that the oxide film on the Ti alloy surface is highly susceptible to attack in the acidic and deaerated PBS solution with H_2_O_2_ and BSA. In this solution high current densities (order of 10^−3^ A/cm^2^) were measured which are not related to passive film on the surface. Acidic and dearated conditions are typical of occluded zones, that is crevice (low access of oxygen and low pH), and H_2_O_2_ can be generated by infections (production of hydrogen peroxide). These results also suggest that when oxygen easily accesses Ti alloys surface, it helps surface re-passivation.

Pieretti et al. [19] evaluated the electrochemical behavior of ASTM F139 austenitic stainless steel in near neutral PBS solutions, either with or without albumin. Addition of albumin (BSA) to the environment resulted in ennoblement of open circuit potential (OCP), suggesting a beneficial effect of BSA on the ASTM F139 stainless steel surface in neutral environments. Barão et al. [45] evaluated the corrosion behavior of Ti-cp and Ti-6Al-4V alloy in artificial saliva medium at different pH (3.0, 6.5 and 9.0) by electrochemical impedance spectroscopy (EIS) and observed higher corrosion rates in the acid (pH 3.0) environment. However, pitting was not observed under any of the conditions tested.

Cheng and Roscoe [20] investigated the effects of calcium, BSA and fibrinogen on the cp-Ti corrosion behavior in phosphate buffer solution (pH 7.4) at 37°C using electrochemical techniques, specifically open circuit potential (OCP) measurements, electrochemical impedance spectroscopy and polarization tests. It was found that BSA addition caused decrease in OCP relatively to the solution without BSA. Lower impedances were also associated to the BSA containing solution suggesting a harmful effect in neutral PBS solution. It was also found that BSA addition resulted in decreased cathodic currents but increased anodic currents indicative of catalytic effect on oxidation reactions at the surface. Addition of protein (BSA) to PBS shifted OCP in the negative direction supporting its depolarization effect of the anodic reaction increasing its kinetic. In our study, the results supported those obtained by Barão et al. [45] showing that BSA had a detrimental effect on the corrosion resistance of the Ti alloy.

The present study isolated the effect of inflammatory conditions on titanium surfaces independent of bacterial biofilms to avoid confounding by bacterial-titanium interactions [46]. It is reasonably expected that the presence of bacterial biofilm will aggravate titanium corrosion based on existing data [7] and future work needs to build upon the current data to specifically assess its effect on titanium corrosion resistance. Previously, Rodrigues et al. [7] exposed Ti dental implants to monocultures of *S*. *mutans* (pH 5) *in vitro* and found that the protective properties of the surface oxide were altered and associated with increased titanium dissolution. The same findings were made in a more clinically representative multi-species bacterial model that showed that the effect of bacteria on titanium corrosion resistance was due to their interaction with the electrical conductivity of titanium [46]. Importantly, Daubert et al. [18] recently made the important discovery of a bidirectional relationship in titanium-biofilm interaction. They showed that titanium particles in peri-implant plaque shape its structure and composition with a particular enrichment in lactate fermenting taxa that may potentiate the adverse acidic environmental conditions [18]. Thus, the inclusion of bacterial interactions in subsequent investigations becomes critical for the understanding of titanium corrosion resistance in the peri-implant environment.

## Conclusions

In this study peri-implant inflammatory conditions were simulated *in vitro* and extensive electrochemical testing to determine factors affecting corrosion susceptibility of Ti-6Al-4V titanium surfaces were performed in various solutions to investigate the determinants of corrosion resistance in conditions simulating peri-implant inflammation. The conditions that simulate crevice millieus, that is, low availability of oxygen and acid (pH 3), as well as those that simulate peri-implantitis, such as, presence of H_2_O_2_ and acid are prejudicial to the corrosion protection of the Ti alloy surface by weakening the oxide film. Also, the presence of BSA addition (1.0 g/L) in the test environment (H_2_O_2_, pH 3, deaerated) has a harmful effect on the corrosion resistance of the Ti-6Al-4V alloy surface resulting in a strong attack of the passive film.
