# Correction: Determinants of corrosion resistance of Ti-6Al-4V alloy dental implants in an *In Vitro* model of peri-implant inflammation

**DOI:** 10.1371/journal.pone.0217671

**Published:** 2019-05-28

**Authors:** Larissa O. Berbel, Everson do P. Banczek, Ioannis K. Karoussis, Georgios A. Kotsakis, Isolda Costa

The third author’s name is spelled incorrectly. The correct name is: Ioannis K. Karoussis.

The correct citation is: Berbel LO, Banczek EdP, Karoussis IK, Kotsakis GA, Costa I (2019) Determinants of corrosion resistance of Ti-6Al-4V alloy dental implants in an *In Vitro* model of peri-implant inflammation. PLoS ONE 14(1): e0210530. https://doi.org/10.1371/journal.pone.0210530
